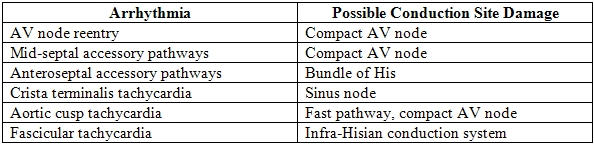# Difficulties with Ablation for Arrhythmias in Children

**Published:** 2008-05-01

**Authors:** Samuel J Asirvatham

**Affiliations:** Division of Cardiovascular Diseases, Department of Internal Medicine, Mayo Clinic, Rochester, Minnesota

**Keywords:** pediatric ablation, electrophysiology, radiofrequency, accessory pathway, AV node reentry

## Abstract

Radiofrequency ablation procedures in children present unique challenges for the electrophysiologist. At times, obtaining vascular access to reach the heart is a problem. If this first step is accomplished, the small size of the child's heart, arrhythmias relatively unique to the pediatric population, and the presence of congenital heart disease add to the complexity.

In this manuscript, a review of commonly encountered problems and suggested solutions based on practice are presented. Precise mapping of the arrhythmogenic substrate, techniques to access excluded portions of the atrium from prior surgery, and the basis for electrophysiology maneuvers important in pediatric ablation are highlighted.

## Introduction

Catheter ablation has revolutionized the management of cardiac arrhythmias in patients. Since the introduction of radiofrequency energy ablation in the late 80s, the major advances and utility for this technology has been in the adult population.

Pediatric electrophysiologists initially had to modify existing technology and tools to serve the needs of the child with otherwise eminently treatable and often curable arrhythmias. Even with such modifications, smaller patients, particularly with abnormal anatomy, were challenging in terms of application of catheter-based ablation [1-3].

Over the last decade, however, with improved understanding of the differences in the risks and benefits of catheter ablation in children and the design of specific catheters and non-radiofrequency-based ablation as well as more advanced imaging and mapping systems, present ablation results in adults are not significantly different from that in children [4]. In particular, the use of point-to-point three-dimensionally rendered electroanatomic maps and cryoablation have facilitated and made ablation safer in children.

Despite these important advances, electrophysiologists sometimes encounter challenges with ablation in children. The purpose of this review is to describe common causes of difficulties in catheter-based ablation and provide an anatomical and electrophysiological framework to understand the reason for this difficulty while suggesting potential methods to solve the specific problem.

It is useful to think of the difficulties with pediatric ablation in two categories. The first involves children with structurally normal hearts but either by virtue of their size [5], difficulties with venous access, particular attention to avoiding collateral damage, or certain arrhythmias unique to children present a challenge. The second category involves common as well as uncommon arrhythmias occurring, however, in the child with congenital heart disease often following surgical correction (palliation or repair).

## Arrhythmias in the Normal Heart

Difficulty with ablation may arise in dealing with relatively common arrhythmias arising in the structurally normal heart. The most common reasons for such difficulties include problems with vascular access and because of the relatively small size of the child's heart, the need for special attention to avoid damage to proximate structures during ablation (collateral damage).

Young children also have a higher likelihood than adults to have certain less common arrhythmias (junctional tachycardia). Normal children's hearts also generally exhibit more rapid conduction than adult hearts and clearly more so than the elderly. These differences create significant modifications on how electrophysiological phenomena observed in children need to be interpreted. For example, very short intraventricular conduction times may give rise to difficulty in distinguishing His bundle and ventricular myocardial capture versus myocardial capture alone when performing para-Hisian pacing [6]. Similarly, when performing electroanatomic mapping (see below), rapid conduction times may give rise to a relatively large area of nearly simultaneous activation (large "red" area) when mapping an automatic tachycardia focus. Discussed in this section are some of the unique challenges facing ablationists who treat arrhythmias in children [7].

### Vascular and Catheter Access Difficulty

**Figure 1:**
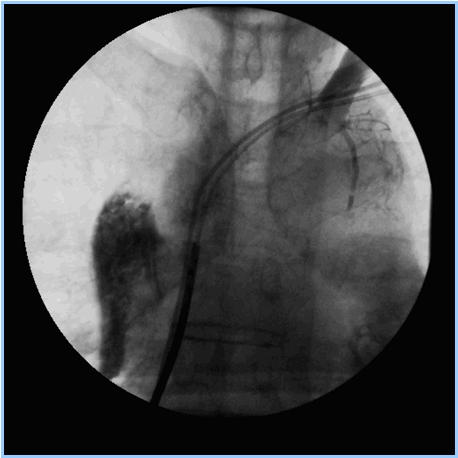
Fluoroscopic image of a large superior vena caval dissection occurring during catheter manipulation following dye injection. SVC dissections are often well tolerated, however, if extravasation of dye is seen, perforation should be diagnosed and treated as a surgical emergency.

Although significant advances in catheter technology have resulted in smaller and more flexible catheters yet retaining torquability and stability during ablation and have greatly reduced the difficulties with catheter placement and vascular access in children, precautions still need to be taken [8]. Dissection of the veins when trying to place catheters is relatively more frequent in children. Carefully delineating the vasculature and using the minimum force required to get catheters to the heart is particularly important for pediatric ablation. Soft guide wires (Glide™) and meticulous attention to technique such as pulling back on the guide wire when advancing a sheath is mandatory to avoid complications. When contrast injection is performed and the results suggest dissection, alternate venous access whenever possible should be sought. Perforation of the major venous vasculature, for example the superior vena cava (Figure 1), represents life-threatening emergencies. If a major vessel perforation is suspected, the perforated sheath or catheter should not be removed but rather kept in situ until the patient is taken for surgical exploration and treatment. A temporizing maneuver that ablationists should be familiar with is placing an angioplasty balloon for tamponade in a major vessel such as the SVC or IVC to control bleeding (Table 1).

Once catheters have been placed in the heart of the child, particular attention must be given when manipulating catheters on the free wall of the right ventricle, anterior wall of the right ventricular outflow tract, and roof of the left atrium as these sites may have a paucity of musculature making perforation risk high [9]. When placing catheters, the fluoroscopic movement of the tip should be watched carefully. As soon as movement of the catheter tip with the heart is noted, no further pressure should be applied (waiting for the catheter to buckle, etc., is an error). Magnet-aided navigation with highly flexible catheters (Stereotaxis™) holds promise for minimizing the risks with catheter manipulation for access, mapping, and ablation in children [10,11].

### Avoiding Collateral Damage

**Figure 2:**
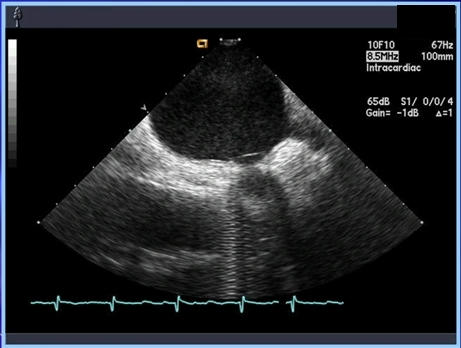
Intracardiac ultrasound image obtained of the left atrium showing a circumferential mapping catheter at the ostium of the left lower pulmonary vein. Accurate knowledge of the pulmonary vein ostium in children is critical and can be difficult to ascertain with standard fluoroscopy.

Even when ablating common arrhythmias with high success rates based on the adult ablation literature, specific precautions are needed in children. For example, ablation of septal pathways or AV node reentry (slow pathway ablation), while sometimes challenging, are generally straightforward in adults. However, with the small size of the heart, specifically the relative proximity of the compact AV node to the posterior septal region in children (smaller CS, smaller subeustachian isthmus), more care is required to avoid inadvertent complete heart block. Another example with contemporary left atrial ablation (atrial fibrillation, left atrial flutters, left atrial tachycardia, etc.) is the extreme caution that is required to prevent damage to contiguous structures such as the pulmonary veins (Figure 2), esophagus, and arterial system. Finally, when ablating outflow tract tachycardias, particularly in the posterior portion of the right ventricular outflow tract in pediatric patients, care to avoid damage to the left main coronary artery is necessary (Table 2).

#### Arterial Damage

The coronary arteries can be damaged during pediatric ablation in several situations, and the ablationists must be cognizant of this possibility and adapt the procedure suitably to avoid potentially devastating sequelae [12].

**Figure 3:**
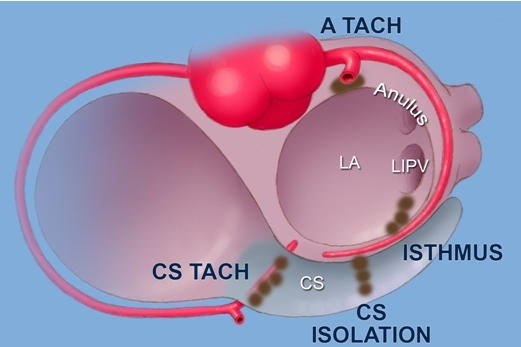
Diagrammatic representation of ablation sites which may involve injury to the major coronary arterial system. Septal mitral annular tachycardias may result in injury to the proximal circumflex coronary artery, whereas the more distal portions of this artery can be injured during I_c_ or coronary sinus-based ablation (see text for details).

**Figure 4:**
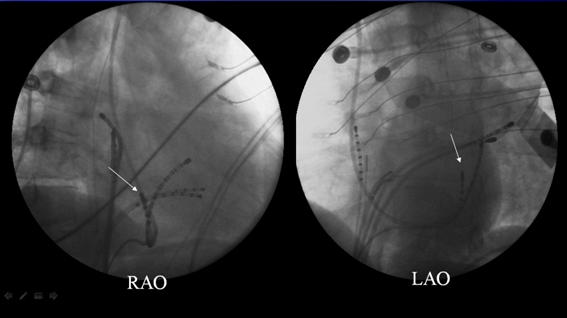
Right anterior oblique (left panel) and left anterior oblique (right panel) projections of catheter (arrow) positioned in the vein of Marshall. Note in the RAO projection, the ablation tip is always kept atrial (posterior) to the body of the coronary sinus. Failure to position the catheter in this manner may result in inadvertent injury to the circumflex coronary artery.

- *Mitral annular tachycardia:* A not infrequent site of automatic atrial tachycardia is in the anterior mitral annulus and now increasingly recognized in the noncoronary cusp of the aortic valve (Figure 3) [13]. Detailed mapping, specifically of the noncoronary cusp and mitral annulus, should first be undertaken preferably with a three-dimensional mapping system. Once the exact site planned for energy delivery is ascertained, a coronary angiogram is prudent to perform and RF ablation energy delivered if the site of tachycardia origin is at least 5-10 mm away from the nearest coronary artery.
- *I_c_ line:* Either as part of a pulmonary vein isolation procedure or specifically for left atrial flutter, an ablation line may need to be done between the left lower pulmonary vein and the mitral annulus. Because of the relative thickness of the myocardium at this site, higher energy/higher temperature ablation is frequently required. In addition, because of the numerous connections between the coronary sinus and left atrium, targeted ablation within the coronary sinus or vein of Marshall is often needed to complete ablation.
  Because of the proximity of the left circumflex artery at this location, care must be taken to avoid damage to this artery. The author's preference is to ablate only until the local electrogram diminishes or becomes fragmented and not continue high energy delivery at these locations. Further, when ablating in the vein of Marshall, the operator should continuously view the RAO projection (Figure 4) to make sure that the ablation energy is delivered atrial to the body of the coronary sinus. At this location, the circumflex artery is almost always ventricular to the coronary sinus. Further, when energy delivery is now continued up to the mitral annulus to anchor the lesion, again ablation only until either bidirectional conduction block occurs or local electrograms at each site have either become split as widely-spaced double potentials or fragmented signals should be done.
- *Coronary sinus isolation or ostial ablation:* Posteroseptal accessory pathways may involve the musculature of the middle cardiac vein. Ablation approaches in this situation in children include isolation of the middle cardiac vein, isolation of the coronary sinus musculature, or ablation at the site of the connection between the middle cardiac vein musculature and ventricles. Each of these approaches have relative merits and demerits, but the main issue to consider is the potential for damaging the posterior descending artery or more commonly branches of the right coronary artery going to the posterolateral wall. In the author's approach, the catheter is first placed at the site of pathway potential or connection between the middle cardiac vein musculature and ventricle (ventricular insertion). A coronary angiogram has been performed and ablation energy delivery if a significant artery is greater than 5 mm away from the ablation site. If arterial proximity is noted, then isolation of the middle cardiac vein ostium is considered again after performing coronary angiography to exclude arterial proximity at this site as well. If neither site is favorable for ablation, then an approach with intravascular ultrasound being placed in the coronary artery to monitor for arterial damage during ablation or an attempt with cryo-energy or isolating the coronary sinus musculature is undertaken.

#### The Esophagus and Vagus Nerve

Ablation in the posterior left atrium is increasingly used in the pediatric population. Linear ablation as part of management of atypical atrial flutter and persistent atrial fibrillation as well as for posterior accessory pathway ablation involves energy delivery in this location. In children, extreme caution should be used to avoid damage to structures behind the left atrium. Thermal damage to the esophagus can be devastating with atrial esophageal fistula formation occurring. The vagus nerve also courses either directly or through its plexus anterior to the esophagus and immediately posterior to the left atrium [14].

**Figure 5:**
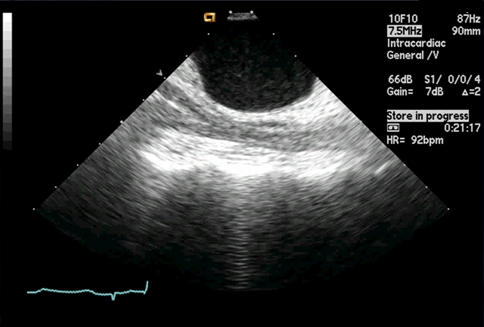
Linear phased-array intracardiac ultrasound image obtained from the right atrium of the posterior left atrial wall and adjacent to the esophagus. The outline of the esophagus can be seen just posterior to the left atrium at this site. Separating these two structures is the oblique sinus or the pericardium. If effusion or tissue changes (increased echogenicity) are noted in the oblique sinus, energy delivery must be stopped and ablation at another site or cryoablation should be considered.

The ablationist must have an approach to avoid thermal damage to the esophagus. The author's preference is utilization of intracardiac echocardiography to visualize exactly the catheter position in relation to the esophagus (Figure 5). Separating the anterior wall of the esophagus from the posterior left atrium is a small amount of fat in children, branches of the vagus nerve, and the oblique sinus of the pericardium. During ablation, the operator visualizes continuously the esophagus and the oblique sinus. Should effusion in the oblique sinus or increased echogenicity be noted on the anterior wall of the esophagus, ablation energy should be immediately turned off. As always (but particularly so with children in this location), ablation energy should not continue after the local atrial electrogram has diminished or become fractionated. These techniques along with minimizing power delivery in this location will help minimize these dangerous complications.

The clinician should also be aware of the postprocedure symptoms that warn of these untoward sequelae and are summarized below (Table 3).

#### Pulmonary Vein Stenosis

Children are particularly susceptible to pulmonary vein stenosis both because of the relatively small pulmonary vein and second because of the relatively thinner layer of myocardium found at the ostium. Accurate knowledge of the exact location of the pulmonary vein ostium and performing ablation only at least 5 mm atrial to the ostium of the vein should be performed when attempting atrial fibrillation ablation in the pediatric population. The combined use of intracardiac ultrasound, possible realtime CT scanning, and the use of electrophysiological maneuvers (discussed elsewhere [15,16]) should all be used for even the experienced ablationist to be certain of avoiding this complication [17].

### Conduction System

Among the most frequently encountered problems in pediatric ablation is avoiding damage to the normal conduction system with energy delivery [18]. This issue is common to several arrhythmias including atrial tachycardia, AV node reentry, and certain forms of ventricular tachycardia (Table 4).

In addition to exact understanding of the regional anatomy and precise mapping (see below), cryoablation and three-dimensional mapping system use have greatly facilitated pediatric ablation near the conduction system.

#### Cryoablation

The premise of cryoablation that cooling to a certain level (-35ºC) results in transient effects (conduction, automaticity), and further cooling (-75 to -80ºC) will result in permanent effects [19]. Thus, when ablating near the conduction system, if with minimal cooling, the accessory pathway blocks but AV nodal conduction remains normal when ablating a mid-septal pathway, it is likely that further cooling will give the desirable result of loss of preexcitation with continued normal AV nodal conduction, etc [19-22].

#### Three-Dimensional Mapping

Three-dimensional electroanatomic mapping (Carto, Johnson & Johnson, Diamond Bar, CA) or Navex (Diag Corporation, Minnetonka, MN) allows detailed delineation of both the conduction system to be avoided and exact localization of the pathway or automatic focus to be ablated [23,24]. When the three-dimensional map is constructed, typically during tachycardia, the conduction system is labeled (annotated) with multiple points being taken to establish accurate geometry. Superimposed on this map the focus/circuit of the arrhythmia is then established, and when these two regions are compared by the ablationist, a plan can be formulated to maximize the chance of ablating the arrhythmia without conduction system damage [23].

## Unique Arrhythmias in Children

While most arrhythmias that occur in adults are seen in children, a few are worth being particularly familiar with for pediatric ablationists. Junctional tachycardia is not commonly seen in adults and needs to be differentiated from unusual forms of AV node reentry so that the pediatric ablationist can consistently manage this arrhythmia without inadvertent heart block. Other arrhythmias such as orthodromic reciprocating tachycardia can be seen in any age group, but in today's practice, the pediatric interventionalist is likely to encounter them more frequently. Because of the size of the young child's heart and the propensity for collateral damage, ablationists who deal with children must be most conversant with techniques that allow exact localization of an accessory pathway (slant, pathway potential identification) to safely manage these arrhythmias.

### Junctional Tachycardia

**Figure 6:**
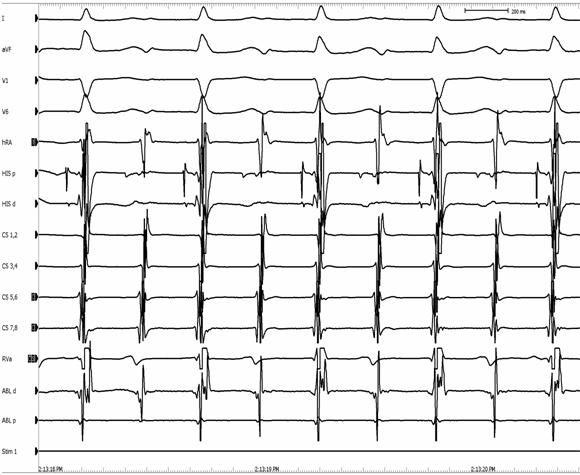
Intracardiac electrogram in a patient with AV node reentry with 2:1 atrioventricular block. Note, atrial electrograms present every other beat without a QRS complex. Block to the ventricle is supra-Hisian in this patient. (CS = coronary sinus; HB = His bundle; hRA = right atrium; RV = right ventricle)

**Figure 7:**
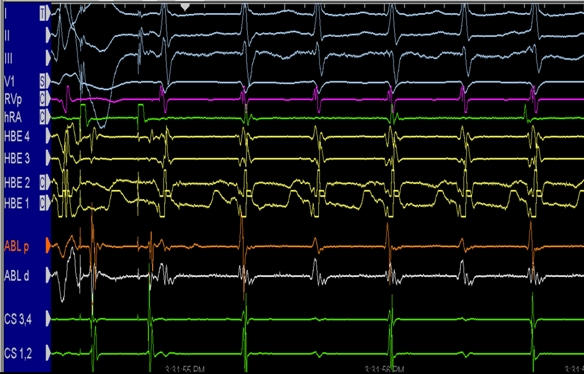
Intracardiac electrograms from a patient with AV node reentry with upper common pathway block. Note, there are more ventricular electrograms than atrial electrograms. The arrhythmia, however, is very regular suggestive of a reentrant circuit (see text for details). (HBE = His bundle electrograms; CS = coronary sinus; ABL = ablation catheter; RV = right ventricle; hRA = right atrium)

**Figure 8:**
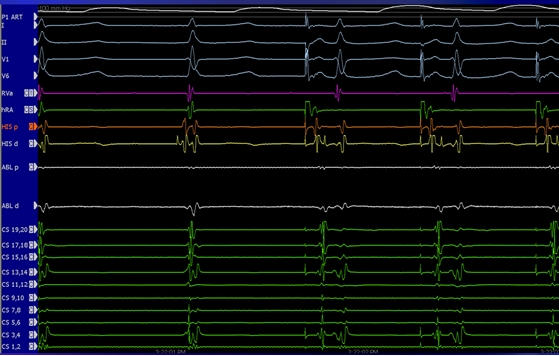
Intracardiac electrograms illustrating atrial pacing that advances the His bundle electrogram with a normal A-H interval. When there is simultaneous ventriculoatrial activation in an arrhythmia, AV node reentry and junctional tachycardia are both possible. However, when late coupled PACs easily advance the next His with a normal A-H interval, AV node reentry can be excluded.

While common in the fetus, junctional tachycardia becomes progressively rarer in the older child and adult. The main cause of difficulty with ablation is diagnosing this arrhythmia as distinct from AV node reentrant tachycardia. At first glance, junctional tachycardia characterized by variable ventriculoatrial association and features of automaticity should be easy to distinguish from AVNRT, a reentrant arrhythmia and expected consistent atrioventricular association [25,26]. In reality, however, these arrhythmias can be exceedingly difficult to distinguish from the other [25,26]. This is because AV node reentry does not necessarily involve a major portion of the AV node and does not involve ventricular myocardium as part of its circuit. Because of this, AV node reentry may present with more As than Vs, more Vs than As, or complete atrioventricular dissociation (Figure 6 and 7). Similarly, junctional tachycardia, typically an automatic rhythm involving the compact AV node or His bundle, does not necessarily bear a consistent relationship with either the atrium or ventricle. A few patterns can help distinguish these arrhythmias. First, premature atrial beats placed during the tachycardia pre-excite the His bundle electrogram with a normal A-H interval (shorter than tachycardia) is diagnostic of junctional tachycardia (Figure 8). This is because in AV node reentry, the excitable gap is small and the PAC, particularly if late coupled, can pre-excite the tachycardia only by antegrade penetration through the slow pathway or when early coupled antegrade penetration through a partially refractory fast pathway, both situations resulting in a relatively long A-H interval. Other patterns of note are that when more As than Vs are seen in AV node reentry, the arrhythmia is still often very stable with little spontaneous variation, suggesting a fixed reentrant circuit rather than abnormal automaticity. The significance of this distinction is that if the ablationist accurately diagnoses the arrhythmia with AV dissociation as AV node reentry, then ablation of the slow pathway at a considerable distance from the compact AV node in the child (3-4 cm) can successfully eliminate the arrhythmia. On the other hand, if junctional tachycardia is diagnosed, cryoablation in regions close to the compact AV node or His bundle will be required with likely increased incidence of heart block [27].

#### Precise Mapping of Accessory Pathways

Perhaps more so than the adult electrophysiologist, the pediatric ablationist must be thoroughly familiar with maneuvers that precisely diagnose accessory pathway location. Such knowledge by itself can minimize the risk of damage to the vasculature or conduction system when these pathways occur in critical locations of the child's heart.

#### The Pathway Potential

The common methods to identify a target for ablation energy delivery to eliminate accessory pathway conduction include fusion of the atrial and ventricular electrograms, the earliest ventricular electrogram during atrial pacing, the earliest atrial electrogram during ventricular pacing (with retrograde conduction), and identification of the pathway potential. While in general any of these methods may be successful, these approaches are not equivalent. The poorest choice for an ablationist is fusion of the atrial and ventricular electrograms. Even when patients do not have an accessory pathway just by virtue of simultaneous activation of adjacent atrial and ventricular myocardium on the annulus (pseudo-interval), fusion may be seen. Once the technique to identify a pathway potential is mastered, this remains the most precise method for pathway ablation.

**Figure 9:**
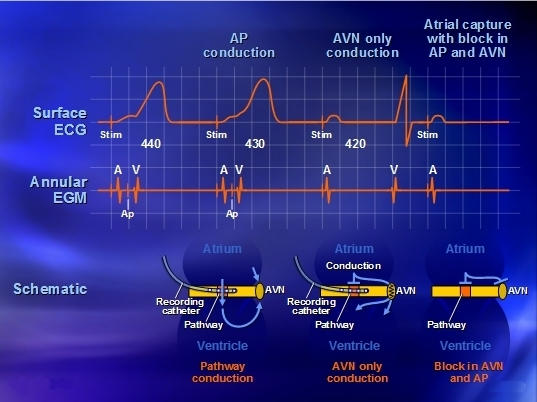
Illustration of the principle for defining an accessory pathway potential (Ap). Note that when atrial pacing at a cycle length of 420 ms occurs, there is loss of preexcitation, although the atrial and ventricular electrograms are retained. Yet, the "candidate" potential is not seen, effectively dissociating this potential from the atrium as well as the ventricle (see text for details).

During atrial pacing, if a candidate potential (suspicious to be a pathway potential) is noted between the atrial and ventricular electrograms during pre-excitation, the ablationist must decipher whether this candidate signal is part of the atrial electrogram, part of the ventricular electrogram, a His bundle recording (in certain situations), or represents the pathway potential. A series of simple maneuvers when accurately interpreted can verify the source of the signal (Figure 9).

1. When the atrium is paced at a rate that produces atrioventricular block, when the candidate electrogram (CE) is still present, it has been effectively dissociated from the ventricle and therefore is not a ventricular electrogram.
2. If with the above maneuver, the CE is no longer seen, then it has been dissociated from the atrium and is not a part of the atrial electrogram.
3. If dissociated from the atrium, then when sensed PVCs are placed during atrial pacing if the CE is unchanged despite changes in the ventricular electrogram, then the CE has also been dissociated from the ventricle.

In most cardiac locations, if the CE has been dissociated from the atrium and the ventricle, a pathway potential can be diagnosed.

**Figure 10:**
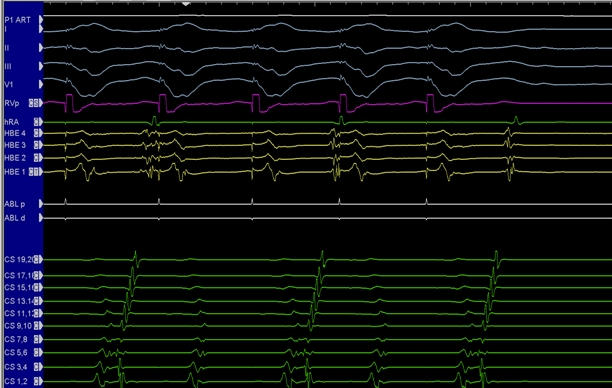
Intracardiac electrograms obtained in a patient with a retrograde conducting left lateral accessory pathway. During ventricular pacing, 2:1 ventriculoatrial conduction is noted. The atrial activation sequence is eccentric. A fragmented electrogram (candidate potentials) is noted in the mid-coronary sinus leads. When ventriculoatrial block occurs, the first component signal remains with the ventricle suggesting that this is part of the ventricular electrogram, whereas the second component does not. Further clarification can be obtained by placing premature atrial beats during ventricular pacing to try and dissociate the atrium from this second signal. The pediatric ablationists may, however, choose to ablate at a site of this signal since it is either a pathway potential or a very early atrial electrogram (see text for details). (CS 1,2 = coronary sinus distal; CS 19,20 = coronary sinus proximal close to the ostium; HBE 1 = His bundle distal recording; HBE 4 = His bundle proximal recording)

Similar logic can be applied for retrograde conducting pathways (Figure 10) and to dissociate the signal from the His bundle or in rare cases from the coronary sinus musculature [28].

#### Pathway Slant

**Figure 11:**
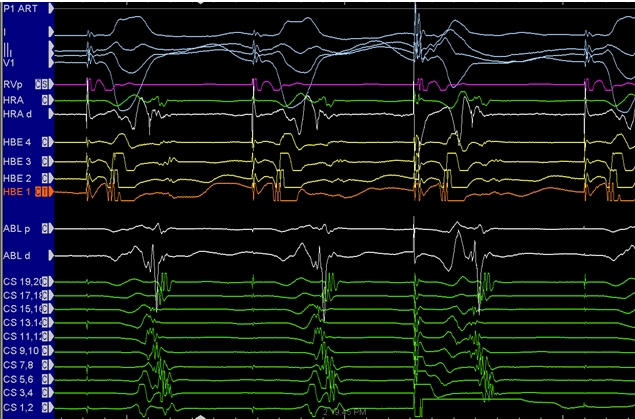
Illustration of pathway slant with intracardiac electrograms during ventricular pacing. The first two beats represent ventricular pacing from the right ventricular apex with the third paced beat from the right ventricle outflow tract region. Note the sudden reversal in the ventricular activation sequence. The pacing rate has not changed, yet the local V-A interval in the mid-coronary sinus is vastly different. With the V-A interval shorter when there is a septal to lateral ventricular wave front, suggests that the ventricular insertion if closer to the septum with the atrial insertion located more laterally (see defining pathway slant in text). (Abbreviations are as in Figure 10.)

Accessory pathways are rarely perpendicular to the atrioventricular annulus (Figure 11). Since annular mapping catheters (coronary sinus catheters) are parallel to the annulus, a unique phenomenon with regard to the atrioventricular interval (with antegrade conduction) occurs when pacing on either side of the accessory pathway and is referred to as pathway slant. One would expect that the atrioventricular interval at the site of the accessory pathway should be fixed and independent of the pacing site (assuming the rate is constant). However, this is not often the case. When an antegrade conducting pathway has its atrial insertion septal to its ventricular insertion at the pathway site, the coronary sinus electrode will record an atrial electrogram while pathway conduction is already occurring (along the slant) resulting in a short A-V interval. When pacing from a lateral location, the atrial electrogram will be inscribed first even before the atrial insertion of the pathway has been activated, thus giving rise to a long A-V interval.

A thorough understanding of this phenomenon is important for two reasons, 1, mapping for an accessory pathway potential with the wave front against the slant (long AV interval) maximizes the chance of seeing the pathway potential, and 2, understanding the actual direction of the slant may allow the ablationist to specifically target the ventricular insertion if that insertion is further away from a sensitive area such as the conduction system or a coronary artery [29].

## Congenital Heart Disease

Among the most significant challenges for an ablationist is managing complex arrhythmias (atypical atrial flutter, scar-related ventricular tachycardia) in a young child with complex congenital heart disease with or without palliative/corrective surgery. A thorough understanding of the relevant anatomy, location of surgical scars, and near perfect interpretation of complex maneuvers (entrainment, arterial potential, etc.) is not only beneficial but often mandatory to success in these situations. A detailed account of each congenital heart disease in terms of anatomy, surgical correction, and arrhythmias encountered is beyond the scope of this review, and the reader is referred elsewhere for these details [28,30-32]. In this paper, we will use the occurrence of atrial arrhythmias following the Fontan procedure to illustrate the commonly encountered problems and suggest solutions.

### Fontan for the Ablationist

Macroreentrant atrial tachycardias commonly affect the quality of life in patients who have previously undergone a Fontan procedure. In approaching these patients, documentation, preferably with a 12-lead electrocardiogram of the clinical arrhythmia is useful as several tachycardias may be inducible in the EP laboratory. The ablationist should be clear on the following issues prior to placing catheters.

**Figure 12:**
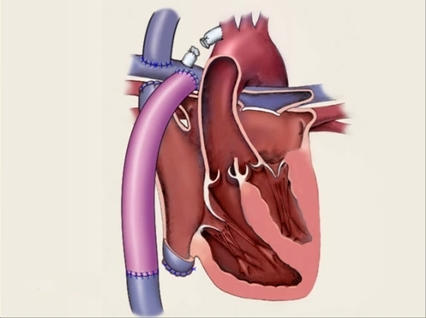
***Univentricular Connections - Fenestrated Fontan Operation:*** Diagrammatic representation of a common type of Fontan procedure in a patient with univentricular AV connections. The ablationist must know the exact type of Fontan procedure, whether a fenestration is present, and where the coronary sinus drains in all cases planned for ablation.

1. What was the exact nature of the Fontan procedure?
  - Can the atria be accessed from both the SVC and IVC?
  - Is there a right atrial appendage to right ventricular outflow tract connection?
  - Is there an intercaval tunnel with a conduit to the right ventricular outflow tract?
2. Where is the coronary sinus located?
3. Is there a fenestration in the tunnel (Figure 12)? [33]
4. Is there a remaining ASD or VSD?

Once the exact anatomy of the specific variant of the Fontan procedure is done in the given patient is known, a plan for catheter access to all parts of the atrium should be made.

**Figure 13:**
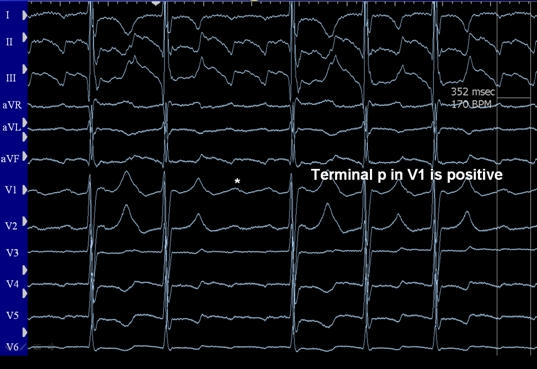
Persistent atrial flutter with terminal positive p-waves in lead V1 suggesting cavo-tricuspid isthmus dependence in a patient status post Fontan procedure. The flutter could be entrained from the cavo-tricuspid isthmus on either side of the Fontan patch (see text for details).

**Figure 14:**
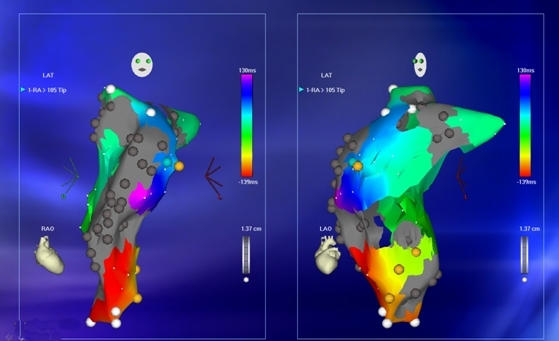
***RA Activation Map in AFL:*** Electroanatomic map in the LAO (left panel) and RAO (right panel) projections. The activation time mapped in the right atrium alone is significantly less than the cycle length of the flutter suggesting an unmapped portion of the atrium.

**Figure 15:**
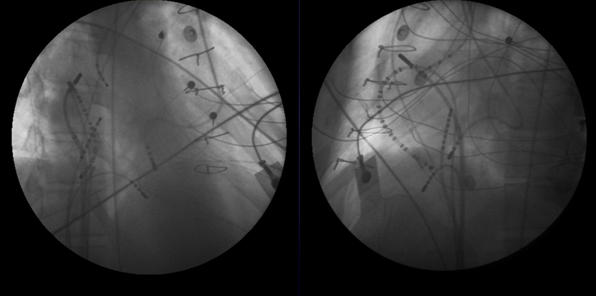
***Retrogarde LV-LA-Neo LA:*** Fluoroscopic images in the RAO (left panel) and LAO (right panel) projections. Note the technique for catheter access of the neo-left atrium in this patient has involved retrograde aortic access then cannulation of the left atrium via the mitral valve and from there across an atrial septal defect into the neo-left atrium.

Figure 13 shows an electrocardiogram consistent with atrial flutter obtained in a patient status post Fontan procedure for tricuspid atresia. The patient had no known fenestration in an intercaval tunnel with a conduit to the right ventricular outflow tract. Via right and left femoral venous access, a detailed map of the right atrium is obtained (Figure 14). Note that the cycle length of the incessant tachycardia was much longer than the total cycle length mapped with the electroanatomic mapping system in the right atrium. This suggests that a significant portion of the circuit is not in the right atrium. Once it has been established that a reentrant tachycardia is present, the entire cycle length of the arrhythmia should be mapped so as to be certain that catheter access and mapping of all potential critical elements of the circuit have been reached (Figure 15).

**Figure 16:**
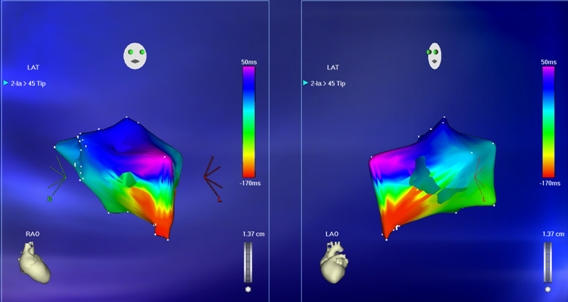
***Neo LA Activation Map in AFL:*** By obtaining catheter access as shown in Figure 15, the neo-left atrium and the portion of the cavo-tricuspid isthmus contained in the neo-left atrium is shown. When the activation times in this map and that of the right atrium shown in Figure 14 are added together, the cycle length of the tachycardia was reproduced.

Patients with this type of Fontan procedure have in effect three atria - the right atria, portions of the original right atria but because of the conduit are now separated from the rest of the right atrium and continuos via a large ASD with the left atrium (neo-left atrium), and the left atrium with the draining pulmonary vein. The neo-left atrium typically houses part of the cavo-tricuspid isthmus particularly the portion just atrial to the tricuspid annulus (Figure 16).

**Figure 17:**
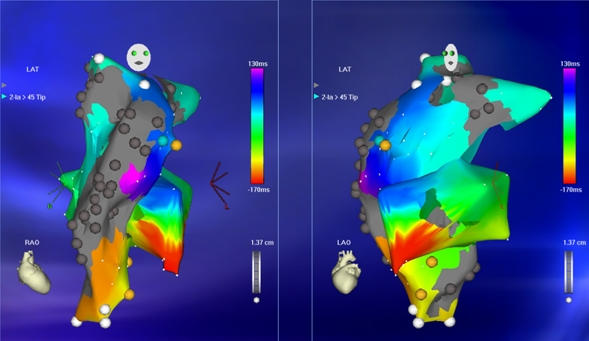
***Combined Activation Map in AFL:*** Combining the activation maps of the right atrium and neo-left atrium defines the entire flutter circuit. Radiofrequency ablation was performed on either side of the Fontan patch in the neo-left atrium and the right atrium (see text for details).

In the patient described, entrainment maneuvers performed on the cavo-tricuspid isthmus both in the neo-left atrium and the right atrium show perfect concealed entrainment. In essence, the cavo-tricuspid isthmus has now been separated into two portions. One, between the tricuspid valve and the conduit and the second from the conduit to the inferior vena cava, with both portions requiring ablation to terminate flutter and create bidirectional conduction block across the cavo-tricuspid isthmus (Figure 17). In this regard, the situation is similar to patients with d-TGA who are status post a mustard or Senning procedure [34]. Here again, the cavo-tricuspid isthmus has separated into two portions but this time by a baffle. In this patient, ablation was performed in the right atrium from the patch to the IVC, and then using retrograde aortic approach with the catheter prolapsed first into the left ventricle and then to the left atrium and through the large ASD into the neo-left atrium, ablation from the tricuspid valve to the patch was completed. The tachycardia terminated and was no longer inducible.

In such situations, the electrophysiologist must be certain of the anatomy involved, equally certain of the critical zone for the reentrant tachycardia, and formulate a plan to obtain catheter access (and contact) to map and ablate the entire circuit and the entire atrium.

### Scar Mapping

An important concept for the pediatric ablationist to be familiar with is that of arrhythmogenic channels being responsible for reentrant tachycardia [35]. The premise for this concept is that normally conducting/healthy atrial tissue is largely irrelevant for tachycardia genesis or maintenance [36]. Similarly, surgical or other (infarction) scars also do not require ablation as no conduction through them is possible. Areas of diseased myocardium with abnormal electrograms but not scars (can be stimulated with pacing) often lying between scar or scar and anatomic obstacles such as the annulus are the culprit slow zones required for reentrant tachycardia maintenance. Since patients with congenital heart disease, prior surgery, and enlarged atria may have multiple tachycardias all of which cannot be mapped in detail and ablated using classical entrainment, scar mapping has largely changed our approach to such cases [37]. When using solely this approach, tachycardia is not induced a detailed bipolar (or unipolar) voltage map of the entire atrial chamber with scar and anatomical obstacles annotated is created. All regions of abnormal myocardium between scars or scars and the annulus, etc., are ablated in an attempt to leave "no channels behind". Often, however, a hybrid approach with detailed mapping of what appeared to be the clinical tachycardia is performed, and in addition, a substrate-based (channel ablation) ablation is also performed [23].

## Summary

Difficulty with ablation for arrhythmias in children may arise for a variety of reasons. Vascular access simply to get catheters to the heart by itself may be a challenge. In the presence of congenital heart disease, accurate knowledge of the anatomy, exact surgical correction, and precise diagnosis of the arrhythmia circuit and neighboring substrate are needed. Even in patients with normal cardiac anatomy, ablation in children can be challenging because of certain arrhythmias (junctional tachycardia and the need to avoid collateral damage) [4]. Avoiding complications with inadvertent ablation of the arteries, conduction system, or pulmonary veins, etc., is best done with first appreciating exact anatomic relationships and then using all maneuvers that allow precise localization of the arrhythmogenic substrate to minimize energy delivery without compromising results.

## Figures and Tables

**Table 1:**
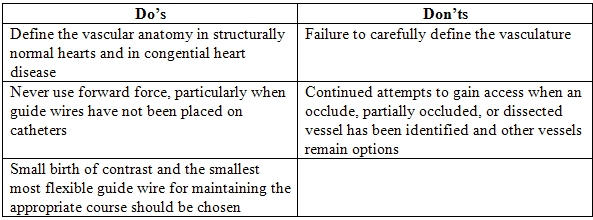
Minimizing Vasculature Damage with Pediatric Ablation

**Table 2:**
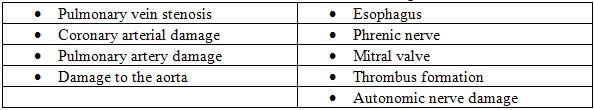
Pediatric Complications of Atrial Fibrillation Ablation

**Table 3:**
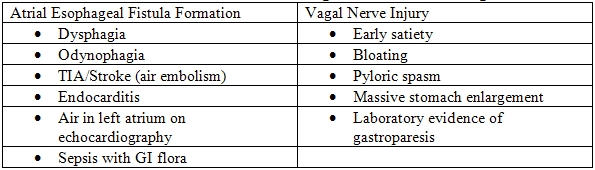
Clinical Signs of Collateral Damage with Posterior Left Atrial Ablation

**Table 4:**